# Treatment device for neonatal birth asphyxia related Hypoxic Ischemic Encephalopathy

**DOI:** 10.1186/s12887-021-02970-z

**Published:** 2021-11-03

**Authors:** Rediet Zewdie, Lidet Getachew, Geremew Dubele, Ababo Oluma, Gedion Israel, Kokeb Dese, Gizeaddis Lamesgin Simegn

**Affiliations:** 1 School of Biomedical Engineering, Jimma Institute of Technology, Jimma University, Jimma, Ethiopia; 2 Bilham Pharmaceutical Private Limited Company, Addis Ababa, Ethiopia; 3 Medicure Medicine and Medical Device Importer, Addis Ababa, Ethiopia; 4 Wolaita Sodo University Teaching Referral Hospital, Wolaita Sodo, Ethiopia

**Keywords:** Birth asphyxia, Cooling therapy, Hypoxic Ischemic Encephalopathy, HIE, Therapeutic Hypothermia

## Abstract

**Background:**

Birth asphyxia is a leading cause of neonatal brain injury, morbidity, and mortality globally. It leads to a multi-organ dysfunction in the neonate and to a neurological dysfunction called Hypoxic Ischemic Encephalopathy (HIE). Cooling therapy is commonly used to slow or stop the damaging effects of birth asphyxia. However, most of the cooling devices used in the healthcare facility do not have a rewarming functionality after cooling therapy. A separate rewarming device, usually a radiant warmer or incubator is used to rewarm the infant after therapy, causing additional burden to the healthcare system and infant families. The objective of this project was, therefore, to design and develop a cost-effective and efficient total body cooling and rewarming device.

**Methods:**

Our design includes two water reservoirs that operate by pumping cold and warm sterile water to a mattress. After decreasing the infant’s core body temperature to 33.5 °C, the system is designed to maintain it for 72 h. Feedback for temperature regulation is provided by the rectal and mattress temperature sensors. Once the cooling therapy is completed, the system again rewarms the water inside the mattress and gradually increases the neonate temperature to 36.5–37 °C. The water temperature sensors’ effectiveness was evaluated by adding 1000 ml of water to the reservoir and cooling and warming to the required level of temperature using Peltier. Then a digital thermometer was used as a gold standard to compare with the sensor’s readings. This was performed for five iterations.

**Results:**

The prototype was built and gone through different tests and iterations. The proposed device was tested for accuracy, cost-effectiveness and easy to use. Ninety-three point two percent accuracy has been achieved for temperature sensor measurement, and the prototype was built only with a component cost of less than 200 USD. This is excluding design, manufacturing, and other costs.

**Conclusion:**

A device that can monitor and regulate the neonate core body temperature at the neuroprotective range is designed and developed. This is achieved by continuous monitoring and regulation of the water reservoirs, mattress, and rectal temperatures. The device also allows continuous monitoring of the infant’s body temperature, mattress temperature, reservoir temperature, and pulse rate. The proposed device has the potential to play a significant role in reducing neonatal brain injury and death due to HIE, especially in low resource settings, where the expertise and the means are scarce.

## Background

Birth asphyxia is a medical condition that causes deprivation of oxygen to a newborn infant before and during birth. Hypoxic-ischemic encephalopathy (HIE) is a brain injury caused by the impeded flow of oxygenated blood to a baby’s brain around the time of birth. It is the leading cause of neonatal brain injury, morbidity, and mortality globally [1]. Perinatal asphyxia may affect virtually any organ, but HIE is the most studied clinical condition and that is burdened with the most severe sequelae [2]. HIE can cause impaired blood-brain barrier permeability, energy failure, loss of cellular ion homeostasis, acidosis, increased intracellular calcium, excitotoxicity, free radical-mediated toxicity, growth factor deficiency, and activation inflammatory cascade damaging the immature brain [3]. The degree of neonatal encephalopathy at birth can be categorized into three stages; stages 1, 2, and 3 [4, 5]. Infants who experience moderate HIE have a 10% risk of fatality and those who live have a 30% risk of disabilities [6]. Infants with severe HIE have a 60% risk of fatality, and nearly all of the survivors experience disabilities [5].

Literature shows that birth asphyxia is a universal public health problem with varied significance country-wise. Worldwide, 23% of neonatal deaths and 10% of all deaths in children under 5 years of age are estimated to occur because of birth asphyxia [7–10]. In sub-Saharan Africa, infant deaths account for 38% of global neonatal mortality due to preventable causes including perinatal asphyxia [11].

A careful neurologic examination needs to be performed to diagnose HIE. History of prolonged and difficult labor coupled with a need for significant resuscitation, low Apgar scores, altered sensorium, and early onset seizures usually point towards HIE. There is an increased risk of neonatal encephalopathy if the mother has fever during antepartum or intrapartum period [12]. Imaging modalities including Magnetic Resonance Imaging (MRI), Computerized Tomography (CT), and Ultrasound (US) are also used to diagnose HIE. MRI is the most sensitive imaging modality for detecting hypoxic brain injury in the neonate [13]. CT scans may be helpful to determine focal hemorrhagic lesions or large arterial ischemic strokes. Ultrasound can be useful for excluding hemorrhagic lesions despite its limited utility in the evaluation of hypoxic injury in the term infant [14]. Accurate diagnosis of HIE leads to early and effective treatment.

Body cooling has been proposed as an effective treatment for HIE in many animal and human experiments [15–22]. Experiment on an animal was conducted by Szczygielski J. et al. [19] on selective brain hypothermia, which is applied via a cranial window after decompressive craniotomy and a reduction in posttraumatic structural and functional damage has been observed. However, the study was limited by the small rodent model and short observational period. Horn AR. et al. [18]. used a servo-controlled fan device to cool 10 infants with neonatal encephalopathy. Infants were nursed on a servo-controlled radiant warmer, set to a target of 33.4 °C–33.7 °C. However, shivering in half of the cases with higher fan speeds and a generally undeniable degree of nursing monitoring compared to servo-controlled systems was noticed. Moreover, the proposed method requires a substantial amount of equipment making it complex and expensive for low resource settings. A phase-changing material (PCM) was also proposed as an effective cooling method for hypothermia of asphyxiated newborns by Tran HTT. et.al [22]. A promising method was designed, demonstrated and tested for low-resource settings using PCM mattress even though out-of-range temperature readings were not measured and controlled by their system.

In general, body cooling treatment devices are frequently used to treat neonates with HIE in many developed countries. Cooling devices used in developed countries are costly, requires maintenance, and has recurring costs. Many low-cost cooling techniques are labor-intensive and may result in temperature fluctuations as well as shivering with a potential loss of neuroprotective efficacy [23]. Moreover, a separate rewarming device, usually radiant warmers are used to rewarm the infant after the cooling therapy, causing additional burden to the healthcare system and infant families. This is especially a challenge in many developing countries, where the resources are scarce. In this paper, a safe, cost-effective, and efficient dual whole-body cooling and a rewarming device is proposed for neonatal birth asphyxia related HIE.

## Method

### The proposed design

In this study, we proposed a dual whole-body cooling and rewarming device for neonatal birth asphyxia related HIE. This automatic cooling and warming device have two reservoirs one for cooling and another for warming purpose. The proposed design solution includes temperature sensors, hot and cold-water reservoirs, arduino mega controller, solenoid valves, peltier cooling and warming device, relays, water pumps, mattress, buzzer, and LCD.

**Fig. 1:**
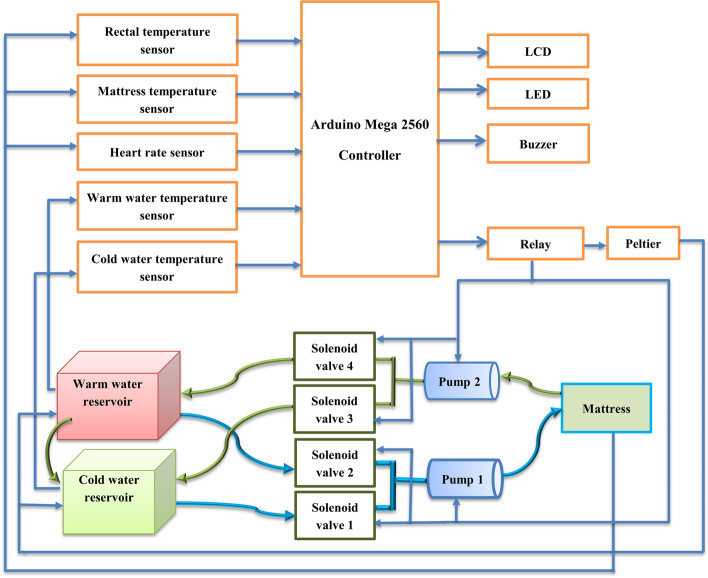
The block diagram of the proposed auto-regulated cooling and rewarming device

This auto-regulated cooling and heating method uses a thermoelectric cooler which is known as peltier device to cool and warm two water reservoirs. The Peltier inside the reservoirs can cool the water as well as work in reverse mode and produce heat to warm the sterile water. The system is designed to automatically control the rotation of the cold and warm water to the specially designed infant mattress based on the rectal and mattress temperature sensor values. Figure 1 demonstrates the functional block diagram of the proposed cooling and rewarming device.

The preparatory phase of the system begins by filling water in the reservoirs in their full capacity. When the power is turned ON, the system powers up the cooling system in reservoir one and at the same time it powers up the heating system in reservoir two. Simultaneously the temperature sensors in both reservoirs will be initiated. Then the user will be prompted to select the temperature values to which the water is to be cooled and warmed. When the coolant reaches the set temperature values, water will be pumped into the neonate mattress.

**Fig. 2:**
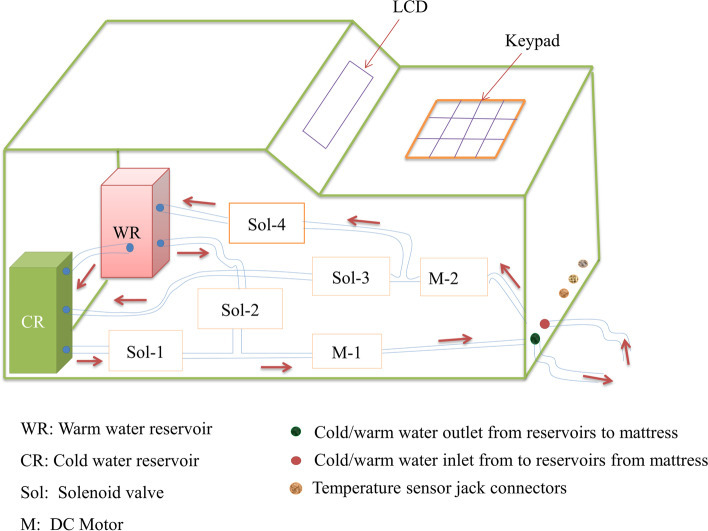
Free-hand sketch of the proposed device and the hydraulic system connections

In the cooling phase, the rectal temperature sensor and mattress water temperature sensor values will be used to monitor the system. When the temperature of water in the neonate’s mattress increases and the temperature of the neonate is above 33.5 °C, cold water will be pumped into the neonate’s mattress from reservoir one and warm water will be sucked from the mattress. The cold and warm water will be rotated in a controlled fashion to sustain the neonate body temperature at 33.5 °C for 72 h. This phase is called the therapeutic phase. The flow of water is controlled by the integration of the DC motor pump and the solenoid valves. The two water pumps are configured to pump and suck water, in and out of the mattress as required. There are a total of four solenoid valves attached to each water pump, used to allow/block water to the mattress (during pumping) and from the mattress (during suction). Each of the solenoid valves are connected to the two tubes attached to the mattress via the water pumps. Figure 2 demonstrates the free-hand sketch of the proposed device and hydraulic system connections.

Out-of-range rectal temperature readings (less than 32.5^0^c or greater than 34.5^0^C) will be detected by the controller during the therapy phase. This will activate the audible alarm and red LED. In addition, a text will be displayed on the LCD informing the caregivers for manual intervention. Red LED will also be turned on. After 72 h of the therapy phase, the rewarming phase begins. A rewarming phase may take 6 to 10-h. In this phase, the neonate will be warmed at a gradual rate of 0.5 °C per hour or less until it reaches a core temperature of 36.5 °C and stabilizes. The earlier cold water in the neonate mattress will be replaced by the warm water. If the rectal temperature increases more than 0.5 °C/hour, the alarm will be activated. The therapy will be completed when the rectal temperature reaches 36.5–37 °C. Cold or warm water will be added to the mattress depending on the target temperature. The sensors result including set temperature, water temperature in cold and hot reservoir, rectal and skin temperature, and mattress temperature are continuously displayed on LCD. A sudden reduction/change of rectal temperature value will be detected and the alarm will be activated followed by a text display “*Check rectal temperature sensor Jack*” on the LCD.

### Temperature regulation phases

**Fig. 3:**
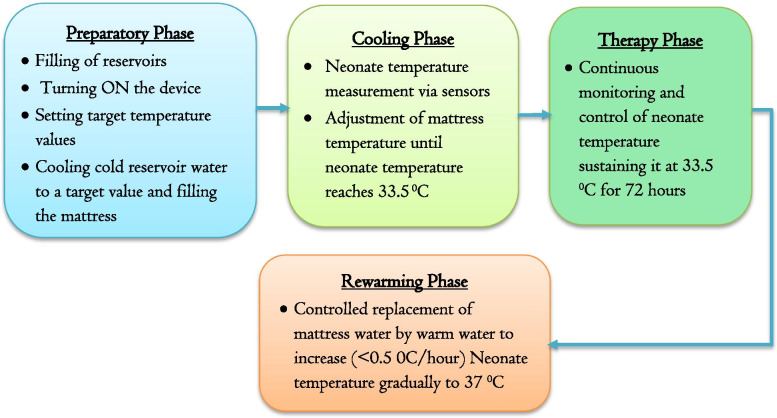
Summary of temperature regulation phases

The proposed system is designed to control regulate temperature for three phases: the preparatory phase, cooling phase, therapy phase, and rewarming phase. In all phases, the rectal temperature of the neonate is continuously monitored and controlled to avoid unstable temperatures which may harm the neonate. Figure 3 demonstrates the four temperature regulation phases and major controlled activities.

#### The preparatory phase

The preparatory phase begins by filling water in the reservoirs until their capacity. In this phase, the coolant will be made ready to be applied to the neonate for therapy. During this phase, initially, the device will be powered by turning on the controller and the temperature sensors for the warm and cold reservoir. The temperature values detected by the sensor will then be displayed on the LCD. Then the system prompts the user to “Enter” the temperatures to which the water is to be cooled and warmed (desired temperature of the coolant and desired temperature of warmer). This will be followed by the cooling and warming of the water in the chambers until the target temperature values are reached in the reservoirs. When the temperature of the coolant reaches the target value, the microcontroller commands the cooling system to stop cooling, powers up the DC motor pump 1, and turns ON solenoid valve 1 using the relay, simultaneously, allowing the flow of coolant liquid into the neonate mattress. After the mattress is filled, the microcontroller sends a command to turn OFF the DC motor pump and solenoid valve 1.

#### The cooling phase

This phase is a preparatory step for the therapy phase. The initial temperature of the neonate will be measured and the mattress temperature will be adjusted accordingly. Initially, the jack of the rectal temperature sensor will be connected to the device and the microcontroller will power up the rectal temperature sensor. Measure rectal temperature value will be displayed on the LCD. If the temperature of the neonate is still above 33.5 °C, the water from the cold reservoir will be pumped into the mattress until the required temperature is acquired.

#### The therapy phase

This phase starts when the temperature of the neonate reaches 33.5 °C. In this phase, the temperature readings will be continuously monitored and controlled. The required rectal temperature value (33.5 °C) will be sustained for the required duration (72 h). If the rectal temperature is less than 32.5 °C during the therapy phase, the microcontroller will activate the buzzer and a text “Over Cooling” will be displayed on the LCD. Simultaneously, the existing cold water in the neonate’s mattress will be replaced by warm water. The process continues until a rectal temperature of 33.5 °C is achieved. On the other hand, if the rectal temperature is greater than 34.50 °C^,^ the microcontroller will activate the buzzer and a text “Over Heating” will be displayed on the LCD. Simultaneously, cold water will be released automatically to the mattress to manage the temperature. The existing warm water will be then removed from the neonate’s mattress until the target temperature is acquired.

#### The rewarming phase

After 72 h of the therapy phase, the microcontroller will activate the DC motor pump and solenoid valves simultaneously, for rewarming. The existing cold water in the neonate mattress will be replaced by the warm water until a target temperature of 37 °C is acquired. A rectal temperature increment above 0.5 °C/hour will be detected and automatically reduced by the controller.

### Materials used

Table 1 demonstrates the materials and their specifications used to construct the prototype.

**Table 1:** List of materials and specifications used to construct the prototype

S. No	Items	Specification
1	Peltier	12 V DC
2	Fan	12 V DC
3	Arduino	Arduino Mega
4	DC motor	12 V
5	Solenoid valve	12 V
6	Mattress	Plastic
7	Power supply	12 V
8	Water temperature sensor	DS18B20
9	Skin temperature sensors	LM35
10	LCD	16 × 2
11	Reservoir	Plastic
12	Relay	12v
13	Buzzer	Buzzer
14	LED	16 × 2
15	Keypad	4 × 4
16	Heat sink	Al heat sink
17	Tubes	PVC

## Results

### Simulation result

**Fig. 4:**
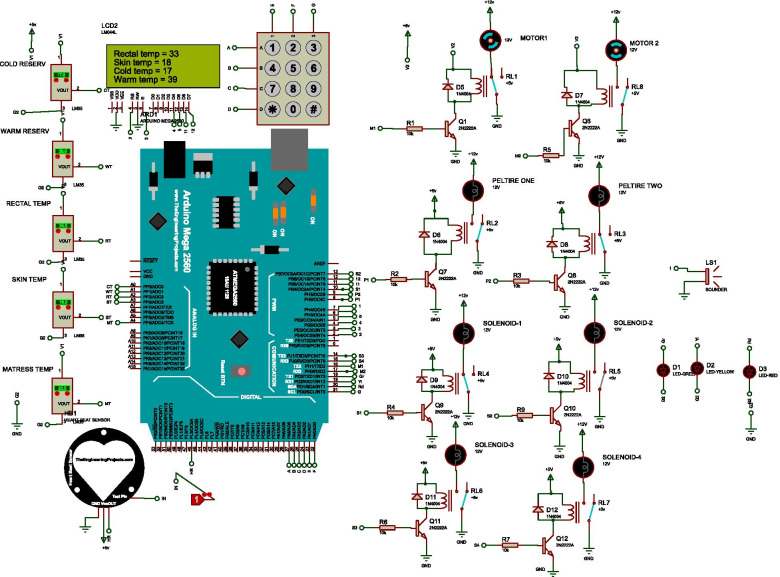
Simulation of the autoregulated cooling and warming system. The proteus connection was done in wireless mode

The design was simulated using Proetus Simulation software and Arduino IDE prior to prototype construction and real testing. The inputs used for the simulated system were cold water temperature value from reservoir-1, warm water temperature value from reservoir-2, rectal and skin temperature values, and treatment time. The system works by accepting input from the sensors. Based on the rectal and skin temperature input, water circulates between the reservoirs and the mattress. Rectal and skin temperature, cold and warm reservoir temperatures, and heart rate and mattress temperature values are displayed on the LCD. An alarm was activated when the temperatures are above the target set values. Body and rectal sensors are continuously displayed on LCD. Figure 4 demonstrates the snapshot of the proposed design simulation.

### Prototype iterations

**Fig. 5:**
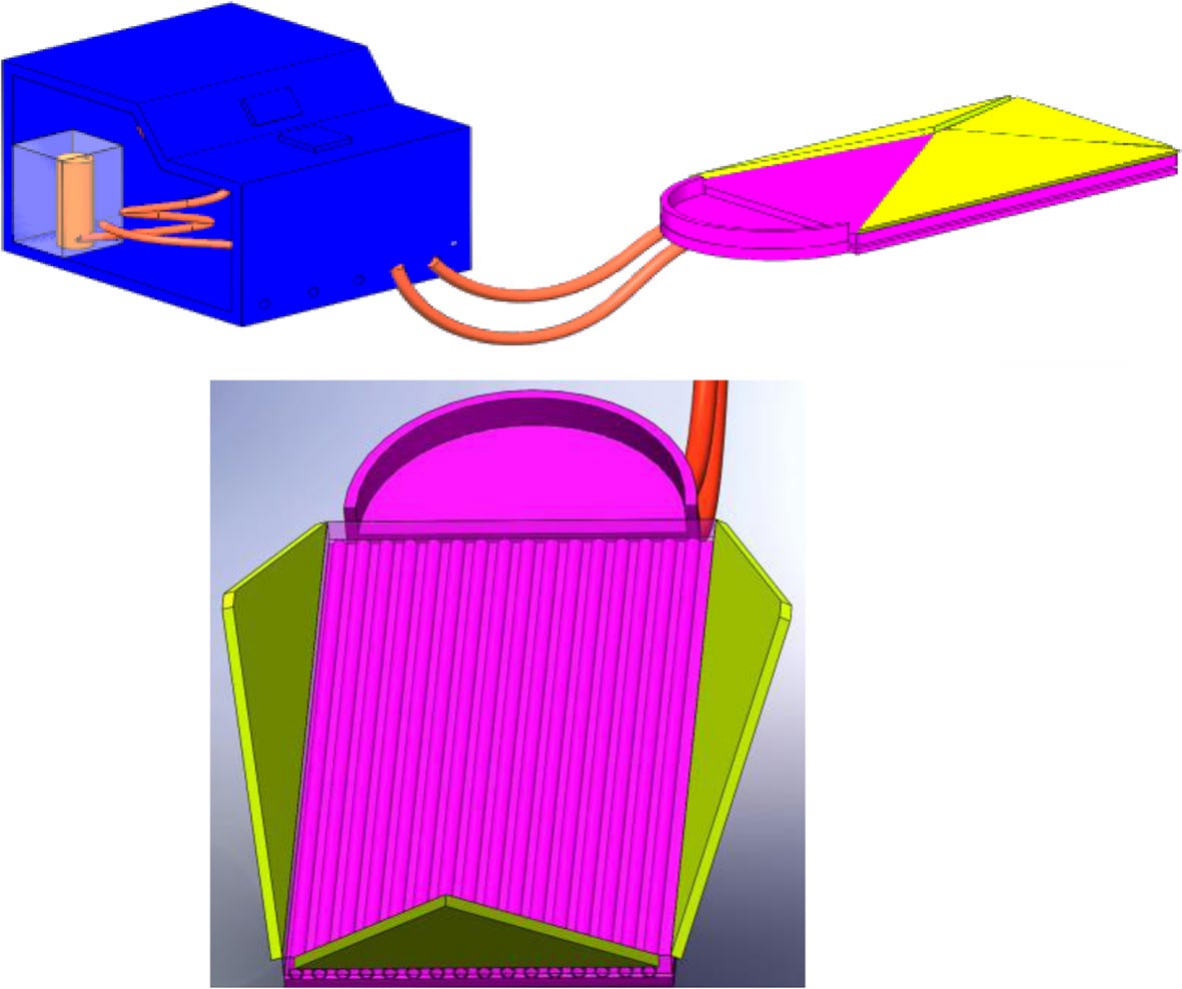
3D design of the proposed device (top) and the mattress (bottom) using AutoCAD

**Fig. 6:**
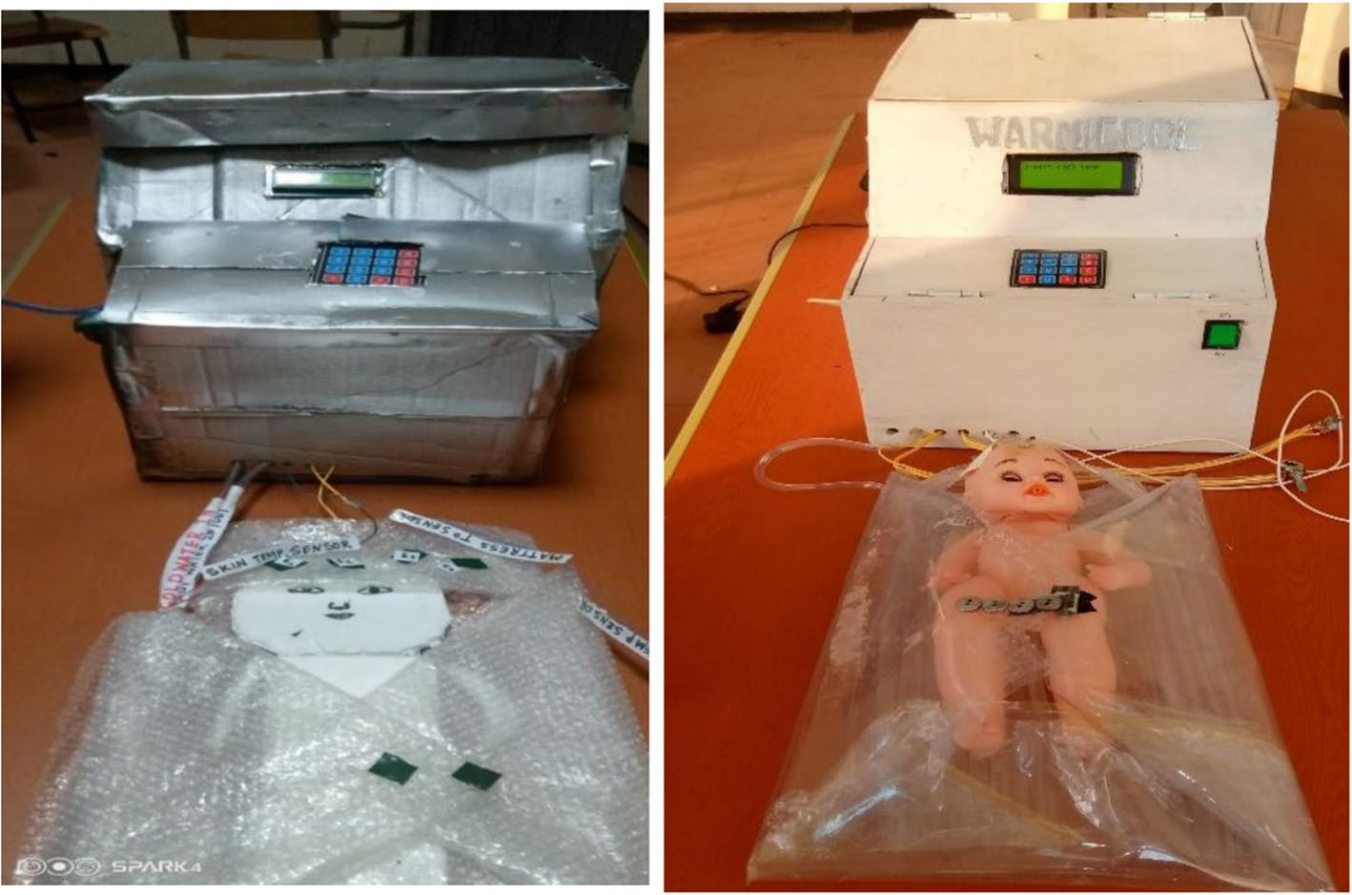
The first and final prototype

Different prototype iterations have been conducted to modify our design. Figure 5 shows the 3D design of the proposed device and the mattress. Figure 6 shows the initial and final constructed prototypes. Initially, the body of the prototype was constructed from waste materials. After initial prototype testing, the whole body of the device was constructed from wood.

### Prototype test results

The design criteria used to design and construct the prototype were accuracy, safety, cost, portability, and easy to use. The tests results were conducted against the design criteria. Accordingly, the accuracy of the prototypes units was checked by performing different tests. The accuracy of water temperature sensors in the reservoirs was tested by adding 1000 ml of water to the reservoir and cooling or warming it to the required level of temperature using Peltier. Then, a digital thermometer was used as a gold standard to compare with the sensor’s readings. Following the same procedure, the accuracy of water temperature in the mattress was also tested. Testing was conducted for five iterations and an average temperature measurement accuracy of 93.2% was acquired. Table 2 demonstrates the testing methods and test results obtained.

**Table 2:** Test methods and test results

S.No.	Design criteria to be tested	Method	Result
1	Temperature measurement Accuracy	By measuring the peltier temperature and mattress temperature sensor readings against a digital thermometer	Accurate 93.2%
2	Electrical Safety	The system and mattress are isolated	Isolated system and emergency switch
3	Cost	Component cost	About 8000 Ethiopian Birr (ETB)
4	Portability	Weight measuring	5 KG
5	Easy to use	Operating procedure	30 min training for Physicians

## Discussion

Birth asphyxia, which is a medical condition resulting from deprivation of oxygen to a newborn infant before and during birth, is a common cause of mental retardation, cerebral palsy, and other neurodevelopmental disorders. It causes neonatal encephalopathy and permanent severe neurologic impairment also known as Hypoxic-ischemic encephalopathy (HIE) HIE is a brain injury caused by the impeded flow of oxygenated blood to a baby’s brain around the time of birth. It is the leading cause of neonatal brain injury, morbidity, and mortality globally.

Although the infant health complexities due to HIE is extremely high in developing countries, health facilities in low resource settings including Ethiopia have no access of proper HIE treatment device.

Neonatal therapeutic hypothermia is used to treat HIE by cooling the baby to about 33.5–34.5 °C degrees-Celsius for 72 h, ideally within 6 h of birth/the oxygen-depriving event. Body cooling treatment devices are frequently used to treat neonates with HIE in many developed countries. However, a separate rewarming device, usually radiant warmers are used to rewarm the infant after the cooling therapy, causing additional burden to the healthcare system and infant families. Moreover, these cooling devices and warming devices are not available or limitedly available in developing countries.

In this paper, we introduced a standalone autoregulated cooling and warming system for neonates with birth asphyxia-related HIE. The device consists of temperature sensors, hot and cold-water reservoirs, Arduino Mega controller, solenoid valves for hot and cold water, Peltier cooling and warming device, relays, water pumps, mattress, buzzer, and LCD. The sensors were used to detect the real-time temperature values from the mattress, skin, and reservoirs. These values are then used by the controller to regulate the temperature of the water in the two reservoirs as required and the flow of water using solenoid valves, relays, DC motor pumps, and Peltier. The real-time temperature readings are continuously displayed in the LCD.

The proposed method was first simulated prior to prototype construction. The sensor readings and the feedback mechanism or regulation of the controller were evaluated using the simulator and the system was modified accordingly. The hydraulic system, the electrical system, and the control mechanism were designed, constructed and tested sequentially, and finally; system unit integration was performed. The body of the final design was constructed from locally available wood material. The design was made to be simple and user-friendly for physicians to easily adapt the proposed system with minimum training. The components used for the construction of the prototype costs less than 200USD (excluding design, manufacturing, and other costs), making it potentially affordable for low resource-settings. The accuracy of the sensors used has been tested against a gold standard and average accuracy of 93.2% was achieved for the temperature readings.

Our proposed solution is unique due, (1) re-warming is performed automatically with a circulation of sterile water from a hot reservoir to the mattress, and there is no need for an additional radiant warmer or incubator for rewarming purposes; (2) the infant whole-body mattress is designed from polyurethanes material which is one of the most versatile plastic; (3) temperature and pulse rate sensors are integrated with the system to help the physicians and nurses monitor the vital signs of the infant without interrupting the treatment; (4) a built-in back-up battery which can be used during transport and electricity failure is integrated with the device.

Since the mattress and the main device are electrically isolated, the proposed design provides a high level of safety. Even though there is no direct contact of water with the neonate’s body, the fluids in the reservoir would be best if changed every 2 weeks. The mattress is made up of a reusable polyurethane material, and it is recommended to clean it using disinfectant after each round of treatment.

We acknowledge that there a was lack of real-world testing of the device. Tests on the animal model should be done before clinical testing and evaluation on humans. In this work, we have demonstrated a potentially effective low-cost cooling device that could, with more evaluation be effective, as a means of treating neonates affected by Hypoxic-ischemic encephalopathy.

## Conclusion

In this work, a device that has the potential to monitor and regulate the neonate core body temperature at the neuroprotective range is designed and developed. Two separate water reservoirs, one for cooling and the other for rewarming using a whole-body mattress were used. The prototype was built and gone through different tests and iterations. The average accuracy of the temperature sensor readings was 93.2% accurate. Our preliminary test results demonstrate that the proposed low-cost device is promising and, with more evaluation, can be used as an effective cooling and rewarming method for a neonate with HIE. This will have a great impact in reducing neonates’ mortality, especially in low resource settings where both the expertise and means are in scarce.

## Data Availability

The datasets used and/or analysed during the current study available from the corresponding author on reasonable request.
